# Determining the Optimal Number of Stimuli per Cranial Site during Transcranial Magnetic Stimulation Mapping

**DOI:** 10.1155/2017/6328569

**Published:** 2017-02-26

**Authors:** Rocco Cavaleri, Siobhan M. Schabrun, Lucy S. Chipchase

**Affiliations:** Brain Rehabilitation and Neuroplasticity Unit, School of Science and Health, Western Sydney University, Campbelltown, NSW 2560, Australia

## Abstract

The delivery of five stimuli to each cranial site is recommended during transcranial magnetic stimulation (TMS) mapping. However, this time-consuming practice restricts the use of TMS mapping beyond the research environment. While reducing the number of stimuli administered to each cranial site may improve efficiency and decrease physiological demand, doing so may also compromise the procedure's validity. Therefore, the aim of this study was to determine the minimum number of stimuli per cranial site required to obtain valid outcomes during TMS mapping. Map volume and centre of gravity (CoG) recordings obtained using five stimuli per cranial site were retrospectively compared to those obtained using one, two, three, and four stimuli per cranial site. For CoG longitude, one stimulus per cranial site produced valid recordings (ICC = 0.91, 95% CI 0.82 to 0.95). However, this outcome is rarely explored in isolation. As two stimuli per cranial site were required to obtain valid CoG latitude (ICC = 0.99, 95% CI 0.99 to 0.99) and map volume (ICC = 0.99, 95% CI 0.99 to 0.99) recordings, it is recommended that a minimum of two stimuli be delivered to each cranial site during TMS mapping in order to obtain valid outcomes.

## 1. Introduction

Transcranial magnetic stimulation (TMS) has been used extensively as a noninvasive tool to explore corticomotor physiology [1, 2]. During TMS, motor evoked potentials (MEPs) are recorded in target musculature in response to electromagnetically induced motor cortex activation [3]. Electromagnetic stimulation is delivered via a coil positioned over the participant's scalp. Stimuli may be applied either at a single cranial site or systematically over a predefined grid, in a process known as “mapping” [4]. Single-site analyses provide information regarding corticomotor excitability, while mapping explores the organisation of cortical territories devoted to muscles within the motor cortex [5, 6].

During nonnavigated TMS mapping, five stimuli are commonly delivered to each site on a 10 × 10 cm grid positioned over the participant's scalp [5]. When using neuronavigation to assist with coil placement, a grid is not necessarily required, but repeated stimuli are often applied to each target site [5]. Averaging the recordings obtained following five stimuli at each cranial site is thought to enhance reliability and account for variations in coil orientation. While this approach has been validated previously, the effect of reducing the number of stimuli per cranial site on mapping outcomes has yet to be completely elucidated [5, 7–9]. This is an important consideration, given that protocols involving the delivery of five stimuli per cranial site are time-consuming, restricting their use beyond the research environment [10]. Further, prolonged TMS assessments have been associated with participant fatigue and discomfort, limiting their utility among clinical populations [10]. Longer assessments may also elicit increased MEP variability, as corticospinal activity has been shown to vary with participant concentration levels [11].

There exists a small amount of literature investigating the validity of TMS mapping protocols with varying numbers of stimuli per cranial site. For instance, it has been demonstrated that five stimuli per cranial site are sufficient to obtain centre of gravity recordings within 2 mm of those obtained when using 20 stimuli per cranial site [9]. While this highlights the importance of appropriate parameter selection, there is currently no consensus regarding the optimal number of stimuli per cranial site for use during traditional TMS mapping. The influence of varying numbers of stimuli on other TMS mapping outcomes, such as map volume, has also not been explored [9, 12]. Alternate mapping techniques, involving “pseudorandom,” rather than systematic, site stimulation have been proposed as a means by which to improve TMS map acquisition times. van de Ruit et al. [10] reported that fewer stimuli were required to generate maps using this approach and that valid maps could be acquired in as little as two minutes. However, the evidence supporting this technique is still developing, and it has only been compared to abridged TMS protocols involving the delivery of three stimuli per cranial site, rather than traditional approaches involving five stimuli per cranial site [10, 12]. As the conventional systematic approach towards TMS mapping continues to be common practice, optimising the efficiency of this technique is an important pursuit.

While reducing the number of stimuli administered to each cranial site during TMS mapping has the potential to improve efficiency and decrease physiological demand, such changes may also compromise the procedure's validity. Despite these considerations, the optimal number of stimuli for use during TMS mapping remains unclear. Therefore, the aim of this study was to determine the minimum number of stimuli per cranial site required to achieve valid outcomes during TMS mapping.

## 2. Experimental Procedures

### 2.1. Study Design

**Figure 1:**
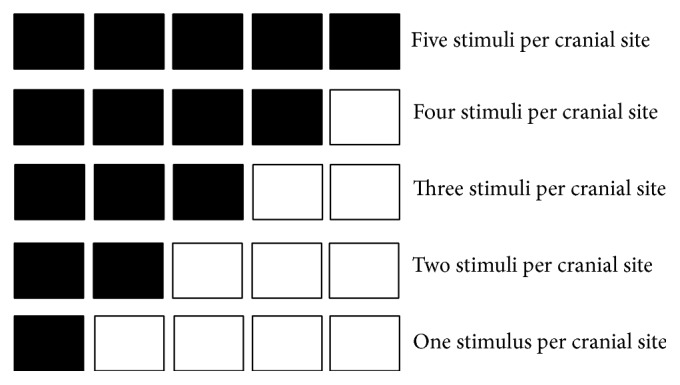
Method used to obtain blocks of varying numbers of stimuli per cranial site.

This study utilised a retrospective analysis to assess the validity of TMS mapping protocols involving varying numbers of stimuli per cranial site. Participant-level data were retrieved from two previously conducted studies [13, 14]. Mapping involving the delivery of five stimuli per cranial site was used as the reference standard [5]. All five stimuli were consecutively delivered to each cranial site. Outcomes obtained using the reference standard were compared to those obtained using one, two, three, and four stimuli per cranial site. Maps for these protocols were obtained by eliminating the required number of stimuli from each original block of five (see Figure 1). A retrospective design enabled a larger sample to be analysed and ensured that experimental procedures were identical across varying numbers of stimuli for each participant. This ensured that the results would not be influenced by factors such as participant arousal or time of day.

Methods were reported in accordance with the TMS-specific checklist developed by Chipchase et al. [15] (score: 24/26, see the Appendix). As this was a retrospective analysis that utilised existing, nonidentifiable data from previous studies, no additional ethical approval was required [16].

### 2.2. Participants

Only data for healthy individuals were considered, as corticomotor activity has been shown to fluctuate significantly within and between TMS mapping sessions involving clinical populations [17]. Participants presenting with acute pain, use of neuroactive drugs (such as anticonvulsants), neurological disorders, or musculoskeletal impairments were therefore excluded. Only baseline data (acquired prior to applying an intervention) were included. Transcranial magnetic stimulation mapping data were retrieved from a total of 34 participants (24 females, 10 males, mean ± SD age of 32 ± 17 years) across the two previously conducted studies. All participants were right hand dominant, and the right hand was tested in all trials. All participants provided data on all outcomes.

### 2.3. Outcome Measures

The following indices of corticospinal plasticity, measured by TMS, were analysed:

- Map volume (in millivolts, mV)
- Centre of gravity latitude (CoG lat) coordinates (cm away from vertex)
- Centre of gravity longitude (CoG long) coordinates (cm away from vertex)

### 2.4. TMS Protocol

Both previous studies employed protocols involving the delivery of five stimuli per cranial site. The same person performed TMS mapping on all participants. All data were obtained from single-pulse, monophasic stimuli delivered using a Magstim 200 stimulator (Magstim Co. Ltd., Dyfed, UK) and a figure-of-eight coil. Neuronavigation was not used in any of the TMS mapping sessions. A five-second interstimulus interval was utilised. The magnetic coil was oriented at a 45° angle in all trials to preferentially induce current in a posterior-to-anterior direction in the cortex [13].

All assessments were performed under resting conditions. The optimal cortical site (“hotspot”) was determined in both studies by identifying the coil position that evoked a maximal peak-to-peak motor evoked potential (MEP). Resting motor threshold (rMT) was defined as the minimum stimulator intensity at which fifty percent of stimuli, applied at the optimal scalp site, evoked a response of at least 0.05 mV (50 *μ*V) within the target muscle [13, 14]. Stimulation intensity was set at 120% of rMT. Electromyography (EMG) recordings were made using silver/silver chloride electrodes, with the recording electrode placed over the FDI muscle belly and the ground electrode placed over the adjacent metacarpophalangeal joint.

### 2.5. Data Processing and Preparation

Peak-to-peak MEP amplitudes were initially analysed in the blocks of five stimuli administered to each cranial site during TMS mapping. This was performed using MATLAB 7 (The MathWorks, USA) [18]. If the mean of the five MEP amplitudes evoked at a particular site exceeded 0.05 mV (50 *μ*V), that site was considered “active” [19, 20]. Active site MEP values were summed to determine the map volume of the target muscle [21]. The centre of gravity (CoG), or amplitude weighted centre of the map, for each muscle was calculated using the formula: CoG = Σ*z*_*i*_*x*_*i*_/Σ*z*_*i*_; Σ*z*_*i*_*y*_*i*_/Σ*z*_*i*_ (where *x*_*i*_ = mediolateral location; *y*_*i*_ = anteroposterior location; and *z*_*i*_ = mean MEP amplitude) [21]. This process was then repeated for the blocks of one, two, three, and four stimuli per cranial site.

### 2.6. Statistical Analyses

This study used TMS mapping involving the delivery of five stimuli per cranial site as the reference standard. Outcomes obtained using five stimuli per cranial site were therefore considered to be the “true” values. For a mapping protocol involving fewer than five stimuli per cranial site to be considered valid, both analyses of variance (or nonparametric equivalents) and intraclass correlation coefficients had to reveal no significant differences between outcomes obtained using that protocol and those obtained using the true value. This strict definition of validity was employed so that recommendations would not be influenced by subjective interpretations of “acceptable” levels of variability. Similar definitions have also been utilised in previous TMS studies [10, 22, 23]. However, mean differences and 95% confidence intervals were also included so that the results would be useful for those with alternate requirements or definitions of validity.

Assumptions of normality and sphericity (equal variance) for parametric analyses were assessed using the Shapiro-Wilk test and Mauchly's test of sphericity, respectively [24]. The Greenhouse-Geisser correction for nonsphericity was applied for data sets that violated the assumption of sphericity [25].

As map volume data failed normality testing, nonparametric analyses, in the form of Friedman's tests and post hoc Sign tests, were employed to compare map volume recordings obtained using five stimuli per cranial site and those obtained using one, two, three, and four stimuli per cranial site [26]. As CoG lat and CoG long data were normally distributed, these outcomes were analysed using one-way repeated measures analyses of variance (ANOVAs) and post hoc ANOVA comparisons. Statistical significance was set at *p* < 0.05, but a Bonferroni correction was applied during all post hoc tests to compensate for multiple comparisons, resulting in a significance level of <0.0125 for these tests.

Absolute intraclass correlation coefficients (ICCs) between recordings obtained using five stimuli per cranial site and those obtained using one, two, three, and four stimuli per cranial site were calculated for each outcome. Intraclass correlation coefficients, including their confidence intervals, were interpreted using the following values: less than 0.50 = poor; 0.50 to 0.65 = moderate; 0.65 to 0.80 = good; and greater than 0.80 = excellent [27]. Thus, for a protocol to be considered valid, the lower boundary of the 95% confidence interval surrounding the ICC between recordings obtained using that protocol and those obtained using five stimuli per cranial site was required to be above 0.80 [27].

## 3. Results

### 3.1. Map Volume

**Figure 2:**
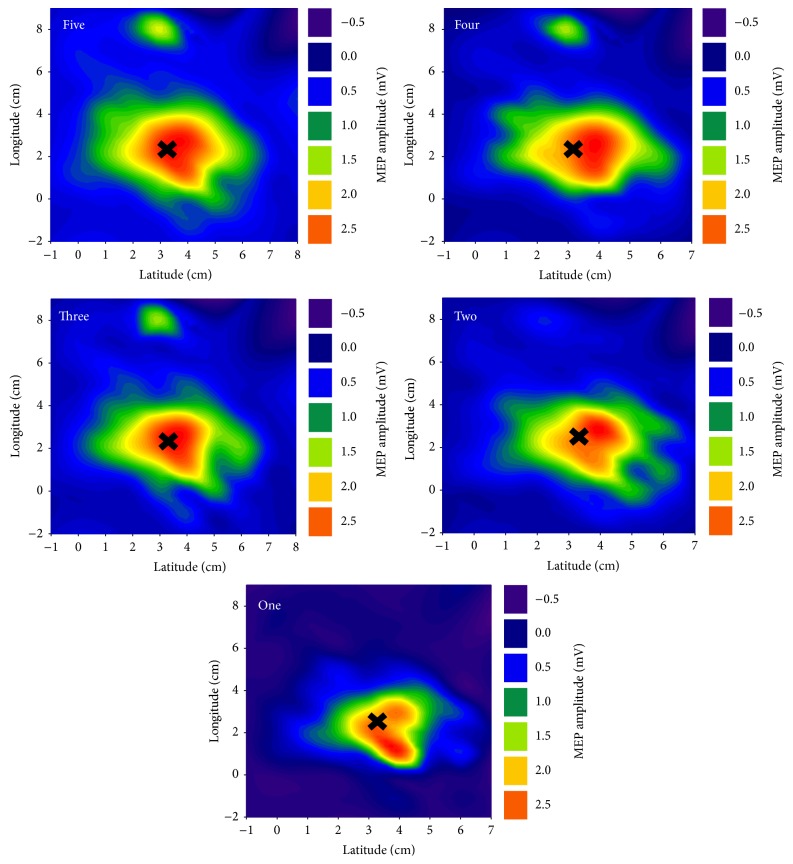
Mean TMS maps for varying numbers of stimuli per cranial site. The black cross (x) highlights the map centre of gravity.

Median (IQR) map volume recordings for one, two, three, four, and five stimuli per cranial site are shown in Table 1. Map volume differed with varying numbers of stimuli per cranial site (*χ*^2^(4) = 27.88, *p* < 0.001). Post hoc testing (conducted with a Bonferroni correction applied, resulting in a significance level set at *p* < 0.0125) revealed that map volume was smaller when five stimuli were delivered than when one stimulus was delivered (*Z* = −3.359, *p* = 0.001). There was no difference in map volume between five stimuli per cranial site and two (*Z* = −0.88, *p* = 0.38), three (*Z* = −2.30, *p* = 0.22), or four (*Z* = −1.95, *p* = 0.05) stimuli per site, suggesting that a minimum of two stimuli per cranial site was required to achieve valid map volume recordings (significance level set at *p* < 0.0125). This was supported by ICC analyses, which revealed that a minimum of two stimuli per cranial site was required for the entire 95% confidence interval surrounding the ICC to be greater than 0.80 (see Table 1). Recordings obtained using only the second stimulus at each site (mean ± SD = 15.32 ± 11.83 mV) were smaller than those obtained using only the first stimulus at each site (mean ± SD = 18.91 ± 12.89 mV, *t*(33) = −10.149, *p* < 0.001).  Figure 2 highlights the differences in map volume and shape between five stimuli per cranial site and one stimulus per cranial site.

### 3.2. Centre of Gravity Latitude

Mean (SD) CoG latitude recordings obtained using one, two, three, four, and five stimuli per cranial site are shown in Table 2. CoG latitude differed with varying numbers of stimuli per cranial site (*F*(1.49,49.14) = 5.15, *p* = 0.02). Post hoc testing (with a Bonferroni correction applied, resulting in a significance level set at *p* < 0.0125) revealed that CoG latitude was smaller when five stimuli were delivered than when one stimulus was delivered (*F*(1.00,33.00) = 8.23, *p* = 0.01). There was no difference in CoG latitude between five stimuli per cranial site and two (*F*(1.00,33.00) = 0.71, *p* = 0.41), three (*F*(1.00,33.00) = 1.90, *p* = 0.18), or four (*F*(1.00,33.00) = 1.66, *p* = 0.21) stimuli per site, suggesting that a minimum of two stimuli per cranial site was required to achieve valid CoG latitude recordings (significance level set at *p* < 0.0125). Intraclass correlation coefficient analyses showed that all of the varying numbers of stimuli produced valid CoG latitude recordings (where the entire 95% confidence interval surrounding the ICC > 0.80). Therefore, two stimuli per cranial site was the minimum number required to satisfy both of the requirements for valid CoG latitude recordings (no significant differences with five stimuli per cranial site on both ANOVA and ICC analyses).

### 3.3. Centre of Gravity Longitude

There were no differences in CoG longitude across the varying numbers of stimuli per cranial site (*F*(2.31,76.26) = 1.92, *p* = 0.15), suggesting that one, two, three, and four stimuli per cranial site all produced valid CoG longitude recordings. This finding was supported by the ICC analyses presented in Table 3.

## 4. Discussion

This study explored the minimum number of stimuli required to achieve valid map volume and centre of gravity (CoG) recordings during TMS mapping. For CoG longitude, one stimulus per cranial site was sufficient to produce valid recordings. However, a minimum of two stimuli per cranial site was required to achieve valid recordings for both CoG latitude and map volume. As CoG longitude is rarely explored in isolation and is typically measured in conjunction with CoG latitude, the results of this study indicate that a minimum of two stimuli can be delivered to each cranial site during TMS mapping in order to achieve valid outcomes when measuring map volume and organisation.

Transcranial magnetic stimulation mapping is a common technique used to explore corticomotor adaptation [1, 2]. However, previously observed variability in coil orientation and MEP recordings during TMS mapping may limit the ability of this procedure to detect subtle physiological changes [7, 23]. While such issues can be addressed by averaging recordings obtained following repeated stimulations, there is a trade-off between accuracy and data acquisition times. Increasing attention is therefore being given to the number of stimuli required to achieve valid TMS outcomes. Currently, the delivery of five consecutive stimuli per cranial site is recommended during TMS mapping [5].

The results of the present study support anecdotal observations that the delivery of five stimuli per cranial site during traditional TMS mapping is inefficient and unnecessary [5]. By implementing the results of the present study and administering two stimuli per cranial site instead of five, investigators may validly reduce data acquisition times. Doing so may result in smaller fluctuations in corticospinal excitability associated with deteriorations in participant arousal and concentration [28]. Reduced data acquisition times would also be beneficial during assessments involving clinical populations, where pain and increased metabolic demands limit adherence to prolonged assessment procedures [29]. However, further research is required to determine if the results of the present study translate to clinical populations.

In this study, map volume recordings obtained using one stimulus per cranial site were larger than those obtained using five stimuli per cranial site. Such findings may be indicative of habituation to repeated stimulation. This decrease in the responsiveness of corticospinal neurons with repeated stimulation is thought to be due to depression of excitatory synaptic activity or an increase in the inhibition of excitatory interneurons [30]. However, decreases in MEP amplitude with repeated stimulation have only been observed at single cranial sites when short interstimulus intervals (one second or less) are employed [10]. While it is possible that longer interstimulus intervals may also influence corticospinal excitability [31, 32], a more likely explanation for this study's findings is that the coil movement required during TMS mapping influenced MEP amplitudes. Unlike observations at a single cranial site, TMS mapping involves systematically moving the stimulation coil from one site to the next, and so recordings may be affected by movement artefact [33–35]. During coil movements, hair follicles may be stimulated and participant arousal may increase, potentially leading to an increase in MEP amplitude with the first stimulus at a particular cranial site [33–36]. Responses may then normalise as the coil is held steady for the remaining stimuli at that location. This notion is supported by the fact that the map volume recordings obtained using only the second stimulus at each site were smaller than those obtained using only the first stimulus at each site.

Despite a rigorous approach towards data collection and synthesis, this study is not without limitations. The exclusion of clinical populations means that the results of this study are only applicable to healthy participants. Likewise, as this study was limited to upper limb musculature, the results may not be generalised to muscles of the lower limb or spine, which have deeper representations within the motor cortex [37]. As neuronavigation was not employed in this study, the findings may not be applicable to clinical procedures involving neuronavigation, such as preoperative motor mapping. While nonnavigated TMS mapping has been shown to be comparable to neuronavigated TMS mapping involving healthy participants, further research is required to determine if the findings of this study may be utilised in clinical contexts [36]. This is particularly important given that even small variations in coil placement can influence MEP recordings and that the utilization of neuronavigation in both clinical and research contexts is increasing [23, 38, 39].

The findings of this study are also only applicable to “traditional,” systematic TMS approaches. Alternate mapping techniques, involving unevenly spaced stimulation grids and “pseudorandom,” rather than systematic, site stimulation have been shown to be reliable when only one stimulus is delivered to each target site [10, 40]. However, the evidence supporting such techniques is limited, and pseudorandom approaches have only been compared to abridged TMS protocols involving the delivery of three stimuli per cranial site. Despite this, it is worth noting that the present study indirectly contributes towards validation of pseudorandom protocols by confirming that TMS mapping involving three stimuli per cranial site (or even two stimuli per cranial site) represents an appropriate reference standard. The findings of the present study therefore appear to be consistent with those involving pseudorandom stimulation and unevenly spaced stimulation grids, even though the latter methods require neuronavigation [40].

Although this study highlights the potential for improving the efficiency of TMS mapping procedures, further research is required. Exploration of lower limb and spinal musculature, including studies involving active rather than resting conditions, is required to determine the optimal number of stimuli per cranial site during TMS mapping involving these regions. A greater emphasis should also be placed upon clinical populations in order to determine if the results of this study are generalizable beyond healthy individuals. Future research should also seek to validate the present study's findings using alternate mapping protocols and grid configurations. For example, while a denser stimulation grid (less than one cm spacing) would increase the number of target sites over a particular area, it could reduce the need for repeated stimulation at those sites and potentially improve the overall accuracy of the motor maps. Similarly, alternate analysis techniques may also influence the optimal number of stimuli per cranial site. Various interpolation and smoothing techniques have been employed during TMS mapping in order to account for within-subject fluctuations in MEP amplitudes and reduce the need for evenly spaced stimulation grids [40]. Future research into such techniques is important, as their utilization would likely influence the minimum number of stimuli required during TMS mapping.

## 5. Conclusions

The results of this study indicate that valid map volume and CoG recordings can be obtained using a minimum of two stimuli per cranial site during TMS mapping of the upper limb. Such findings have the potential to greatly reduce data acquisition times and participant discomfort. Future research should seek to determine if these findings can be applied to clinical populations and lower limb or spinal musculature.

## Figures and Tables

**Table 1:** Map volume data (in mV) for varying numbers of stimuli per cranial site.

Number of stimuli per cranial site	Median (IQR)	Mean difference with reference standard (95% CI)	ICC (95% CI)
One	14.20 (8.18 to 24.07)	2.85 (0.90 to 4.79)	0.90 (0.62–0.96)
Two	12.08 (6.83 to 18.30)	0.11 (−0.38 to 0.60)	0.99 (0.99–0.99)
Three	14.07 (7.09 to 20.34)	0.73 (0.11 to 1.36)	0.99 (0.96–0.99)
Four	12.92 (6.89 to 19.70)	0.37 (0.08 to 0.67)	0.99 (0.99–0.99)
Five	12.52 (6.61 to 18.43)	NA	1.00 (NA)

Key: IQR = interquartile range, ICC = intraclass correlation coefficient with five stimuli per cranial site (the “true” value), CI = confidence interval, and NA = not applicable.

**Table 2:** CoG latitude (in cm away from vertex) for varying numbers of stimuli per cranial site.

Number of stimuli per cranial site	Mean (SD)	Mean difference with reference standard (95% CI)	ICC (95% CI)
One	3.40 (0.85)	0.10 (−0.01 to 0.20)	0.97 (0.92 to 0.99)
Two	3.31 (0.79)	0.01 (−0.02 to 0.04)	0.99 (0.99 to 0.99)
Three	3.32 (0.81)	0.02 (−0.02 to 0.06)	0.99 (0.99 to 0.99)
Four	3.32 (0.81)	0.02 (−0.02 to 0.05)	0.99 (0.99 to 0.99)
Five	3.30 (0.80)	NA	1.00 (NA)

Key: SD = standard deviation, ICC = intraclass correlation coefficient with five stimuli per cranial site (the “true” value), CI = confidence interval, and NA = not applicable.

**Table 3:** CoG longitude (in cm away from vertex) for varying numbers of stimuli per cranial site.

Number of stimuli per cranial site	Mean (SD)	Mean difference with reference standard (95% CI)	ICC (95% CI)
One	2.70 (1.04)	0.06 (−0.02 to 0.14)	0.99 (0.97 to 0.99)
Two	2.64 (1.01)	−0.01 (−0.04 to 0.02)	0.99 (0.99 to 0.99)
Three	2.67 (1.01)	0.02 (−0.01 to 0.06)	0.99 (0.99 to 0.99)
Four	2.66 (1.01)	0.02 (−0.07 to 0.11)	0.99 (0.97 to 0.99)
Five	2.64 (1.03)	NA	1.00 (NA)

Key: SD = standard deviation, ICC = intraclass correlation coefficient with five stimuli per cranial site (the “true” value), CI = confidence interval, and NA = not applicable.

**Table 4:** 

	Controlled
*Participant factors *	
Age of subjects	Y
Gender of subjects	Y
Handedness of subjects	Y
Subjects prescribed medication	Y
Use of CNS active drugs (e.g., anticonvulsants)	Y
Presence of neurological/psychiatric disorders	Y
Any medical conditions	Y
History of specific repetitive motor activity	Y
*Methodological factors*	
Position and contact of EMG electrodes	Y
Amount of contraction of target muscles	Y
Prior motor activity of the muscle to be tested	Y
Relaxation of muscles other than those tested	N
Coil type (size and geometry)	Y
Coil orientation	Y
Direction of induced current in the brain	Y
Coil location and stability	Y
Type of stimulator used (e.g., brand)	Y
Stimulation intensity	Y
Pulse shape (monophasic or biphasic)	Y
Determination of optimal hotspot	Y
The time between MEP trials	Y
Time between days of testing	Y
Subject attention (level of arousal) during testing	N
Method for determining threshold (active/resting)	Y
Number of MEP measures made	Y
Method for determining MEP size during analysis	Y

*Total score/26*	24

Key: Y = yes, N = no, CNS = central nervous system, EMG = electromyography, and MEP = motor evoked potential.

## Appendix

### Checklist for Reporting of TMS Methodology

See Table 4.
